# Memory Formation Shaped by Astroglia

**DOI:** 10.3389/fnint.2015.00056

**Published:** 2015-11-17

**Authors:** Robert Zorec, Anemari Horvat, Nina Vardjan, Alexei Verkhratsky

**Affiliations:** ^1^Laboratory of Neuroendocrinology and Molecular Cell Physiology, Institute of Pathophysiology, Faculty of Medicine, University of LjubljanaLjubljana, Slovenia; ^2^Celica BiomedicalLjubljana, Slovenia; ^3^Faculty of Life Sciences, University of ManchesterManchester, UK; ^4^Achucarro Center for Neuroscience, Ikerbasque, Basque Foundation for ScienceBilbao, Spain; ^5^Department of Neurosciences, University of the Basque CountryLeioa, Spain; ^6^University of Nizhny NovgorodNizhny Novgorod, Russia

**Keywords:** astroglia, memory, shape, metabolism, signaling

## Abstract

Astrocytes, the most heterogeneous glial cells in the central nervous system (CNS), execute a multitude of homeostatic functions and contribute to memory formation. Consolidation of synaptic and systemic memory is a prolonged process and hours are required to form long-term memory. In the past, neurons or their parts have been considered to be the exclusive cellular sites of these processes, however, it has now become evident that astrocytes provide an important and essential contribution to memory formation. Astrocytes participate in the morphological remodeling associated with synaptic plasticity, an energy-demanding process that requires mobilization of glycogen, which, in the CNS, is almost exclusively stored in astrocytes. Synaptic remodeling also involves bidirectional astroglial-neuronal communication supported by astroglial receptors and release of gliosignaling molecules. Astroglia exhibit cytoplasmic excitability that engages second messengers, such as Ca^2+^, for phasic, and cyclic adenosine monophosphate (cAMP), for tonic signal coordination with neuronal processes. The detection of signals by astrocytes and the release of gliosignaling molecules, in particular by vesicle-based mechanisms, occurs with a significant delay after stimulation, orders of magnitude longer than that present in stimulus–secretion coupling in neurons. These particular arrangements position astrocytes as integrators ideally tuned to support time-dependent memory formation.

## Memory Formation Results in Anatomical Changes

Memory is the process of retention and reconstruction of learned (acquired) knowledge. Studies performed in the early 1960s on patients who underwent bilateral medial temporal lobe surgery, recognized the hippocampus as a fundamental region for memory formation (Scoville and Milner, 1957). Subsequently, two distinct memory systems, declarative (explicit) memory for facts and events, for people, places, and objects (“knowing that”) and non-declarative (implicit) memory, the memory for perceptual and motor skills (“knowing how”), have been defined (Dudai and Morris, 2013). Both systems rely on similar, if not identical, mechanisms associated with reinforcement of synaptic transmission, which involve morphological changes at the synapse that outlast memory stabilization (Attardo et al., 2015). This morphology-based mechanism was considered by Cajal (1894); who linked “cerebral gymnastics” (Box 1) with morphological alterations of dendrites and terminals of neurons.

Box 1 **Cerebral Gymnastics and Memory Formation**

“Cerebral gymnastics are not capable of improving the organization of the brain by increasing the number of cells, because it is known that the nerve cells after the embryonic period have lost the property of proliferation; but it can be admitted as very probable that mental exercise leads to a greater development of the dendritic apparatus and of the system of axonal collaterals in the most utilized cerebral regions. In this way, associations already established among certain groups of cells would be notably reinforced by means of the multiplication of the small terminal branches of the dendritic appendages and axonal collaterals; but, in addition, completely new intercellular connections could be established thanks to the new formation of [axonal] collaterals and dendrites.” The Cronian Lecture: La fine structure des centres nerveux. *Proceedings of the Royal Society of London* 55: 444–468, 1984. Translated by DeFelipe J, Jones, E. G. (1988). *Cajal on the Cerebral Cortex*. New York, NY: Oxford University Press. p. 87.

Contemporary views assume that memory formation, although it is an outcome of a myriad of interactive processes, occurs in the form of molecular events at the level of an individual synaptic connection, which is termed synaptic plasticity. These synaptic changes integrate through multiple synaptic connections involving larger neuronal networks, and are finally expressed at the behavioral level (Kandel et al., 2014).

## Memory Formation and Astrocyte Morphology

Micro-anatomical changes that are part of memory formation are not exclusively related to neurons and their parts, but involve non-neuronal cells, which in many areas of the human brain exceed the number of neurons (Azevedo et al., 2009). These non-neuronal cells include astrocytes, an abundant and arguably the most heterogeneous glial cell type in the central nervous system (CNS). It is generally acknowledged that astroglia actively participate in information processing via cytosolic calcium signals (Verkhratsky et al., 1998; Rusakov et al., 2011).

A single astrocyte is intimately associated with many neurons and with their synaptic contacts. A single rat cortical astrocyte enwraps 4–8 neuronal bodies and 300–600 dendrites (Halassa et al., 2007), and astrocytes are in contact with synapses. In the rat hippocampus, an individual astrocyte can cover (by perisynaptic processes) up to 140,000 synapses (Bushong et al., 2002). Human hippocampal astrocytes are substantially larger and a single human astrocyte may be associated with ~2 million synapses (Oberheim et al., 2006). Abundant morphological interactions of astrocytic processes with neurons are not restricted to the hippocampus, being a widespread property of CNS tissue.

Close morphological apposition allows astrocytes to receive signals from the synaptic cleft and feedback by releasing their own signaling molecules. Release of many of these molecules occurs through a secretory pathway that uses cytoplasmic vesicles, which store chemical messengers. On stimulation, the vesicle membrane fuses with the plasmalemma, a process termed regulated exocytosis. The role of secretory vesicles in astrocytes was proposed in 1910 when Nageotte (1910) suggested, based on his microscopic observations, that glial cells (astroglia in particular) act as secretory elements in the CNS. This hypothesis has been confirmed experimentally in the last two decades by identifying vesicular release of gliosignaling molecules, which are often termed gliotransmitters (Vesce et al., 1999; Haydon, 2001; Parpura and Zorec, 2010; Zorec et al., 2012). Although there is some skepticism that this mechanism exists in astroglia (Fujita et al., 2014; Sloan and Barres, 2014), bidirectional astrocyte-neuron signaling is well accepted, and it is generally recognized that vesicle-based mechanisms participate in the heterocellular signaling that occurs at a morphofunctional unit known as the tripartite synapse (Araque et al., 1999; Perea et al., 2009). This bidirectional communication is part of the wider gliocrine system (Vardjan and Zorec, 2015), which reflects the secretory role of astrocytes, which release an extensive number of gliosignaling molecules (Verkhratsky et al., in press). These molecules are largely not involved in synaptic processes but rather regulate various brain functions through “volume” transmission (Vardjan and Zorec, 2015; Zorec et al., 2015). Astroglia-derived signaling molecules are secreted into the extracellular space and are transported throughout the tissue parenchyma to distant places in the CNS, likely taking advantage of the glymphatic convective system (Thrane et al., 2014).

During implicit memory consolidation of Pavlovian threat conditioning, astrocytic processes retract from synapses in the lateral amygdala, allowing these synapses to enlarge, suggesting that contact with astroglial processes opposes synapse growth during memory consolidation (Ostroff et al., 2014). In other words, if astrocytic processes enwrap synapses and the latter need to expand during memory formation, astrocytes may hinder this remodeling, demonstrating how astrocytic structural plasticity enables morphological remodeling of synapses associated with memory formation. Under physiological conditions, including reproduction, sensory stimulation, and learning, astrocytes display a remarkable structural plasticity. Distal astrocytic processes can undergo morphological changes in a matter of minutes, thus modifying the geometry and diffusion properties of the extracellular space and relationships with adjacent neuronal elements, especially with synapses. This type of astroglial plasticity has important functional consequences because it modifies extracellular ionic homeostasis and neurotransmission, thus ultimately modulating neuronal function at the cellular and system levels (Oliet and Piet, 2004; Theodosis et al., 2008). The mechanisms responsible for morphological changes in astrocytes are not known, but these may likely involve adrenergic receptors and generation of second messenger cAMP (Vardjan et al., 2014), which are discussed in the following section.

## Astrocyte Morphology, cAMP and Metabolism

**Figure 1:**
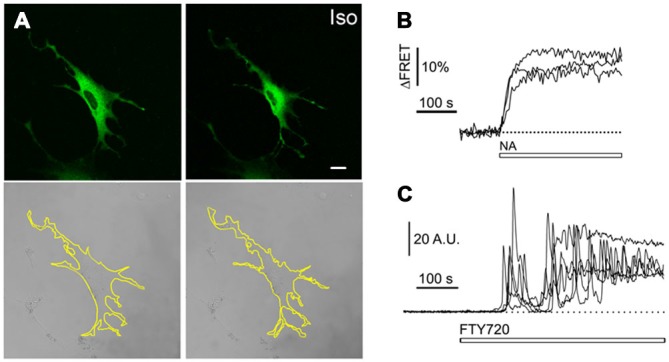
**(A)** Morphological changes in astrocytes (stellation) induced by the β-adrenergic receptor (β-AR) agonist isoprenaline (Iso), which increases cAMP. Green fluorescing astrocytes transfected with the cAMP nanosensor Epac1-camps (top) and their corresponding differential interference contrast images (bottom) before (left) and after 1 μM β-AR agonist isoprenaline (Iso). Note the thinning and elongation of processes indicating astrocyte stellation. Scale bar represents 20 μm. Astrocytes were cultured from rat cortex. Modified from Vardjan et al. (2014) with permission. **(B)** Time course of cytosolic levels of cAMP. Noradrenaline (NA) persistently increases intracellular cAMP levels in astrocytes. Representative time courses of the Epac1-camps (i.e., a Förster resonance energy transfer (FRET)-based cAMP nanosensor) from three cells after the addition of 1 μM NA. Changes in FRET are expressed as percentages relative to the initial values. **(C)** Time course of cytosolic levels of Ca^2+^. The application of fingolimod (FTY720) evokes prolonged transient increases (oscillations). Superimposed time-resolved fluorescence intensity obtained in five cells treated with FTY720 (white bar). The thin dotted line indicates the zero fluorescence level (F_0_). Modified from Vardjan and Zorec (2015) with permission.

Astrocytes are capable of a remarkable morphological plasticity. Astroglial cells *in vitro* have a flattened polygonal appearance, however stimulation of the β-adrenergic cAMP-dependent signaling cascade results in rapid morphological remodeling with astrocytes assuming a stellate morphology with numerous processes (Shain et al., 1987; Bicknell et al., 1989; Hatton et al., 1991; Shao et al., 1994; Won and Oh, 2000; Gharami and Das, 2004; Vardjan et al., 2014). This remodeling occurs within the time frame of memory consolidation (minutes to hours) and involves cytoskeletal reorganization, including the restructuring of actin filaments, microtubules, and intermediate filaments (Goldman and Abramson, 1990; Safavi-Abbasi et al., 2001). An example of this adrenergic receptor/cAMP-mediated morphological remodeling of astrocytes is shown in Figure 1 (Vardjan et al., 2014). Similar morphological plasticity may take place *in vivo* in long-term memory formation because noradrenaline (NA), derived from projections of neurons located in the locus ceruleus (LC), operates as a neuromodulator in Hebbian learning (Johansen et al., 2014). Under similar training conditions, changes in astrocytic shape have indeed been observed (Ostroff et al., 2014). Moreover, the existence of structural-functional changes of the astrocyte-neuron interactions during memory processes have been detected (Lavialle et al., 2011; Bernardinelli et al., 2014; Perez-Alvarez et al., 2014).

Tight association between the synaptic membranes and astrocytes is considered essential for homeostatic control of the synaptic cleft, including rapid removal of the neurotransmitter glutamate (Bergles and Jahr, 1997) and potassium from the extracellular space (Orkand et al., 1966; Verkhratsky and Nedergaard, 2014). Thus, retraction of astrocytic membrane from the synapse during memory formation (Ostroff et al., 2014) may facilitate the spillover of neurotransmitter and thus affect synaptic transmission (Rusakov and Kullmann, 1998). At the same time, memory formation is associated with morphological growth of synaptic elements together with enhanced protein synthesis and rearrangement of receptor proteins, all of which increase the energy consumption (Harris et al., 2012).

How energy substrates, needed for adenosine triphosphate (ATP) synthesis, are delivered to synapses where synaptic plasticity takes place is still an open question. A simple assumption would be that pyruvate is provided to the mitochondria by glycolysis within the neuron. However, the morphology of astrocytes, with extensive end feet plastering blood vessels, is well suited to take up glucose from blood and distribute either glucose itself, or pyruvate or lactate derived from glucose, to astrocytic processes surrounding synapses, possibly by diffusion through gap junctions integrating astroglial syncytia (Rouach et al., 2008). In support of this mechanism, diffusion of glucose within astrocytes is relatively rapid (Kreft et al., 2013) and may well support glucose delivery via interconnected astrocytes *in situ*. Although synapses are the main energy consumers in the brain, glycogen, the only CNS energy storage system, is present mainly, if not exclusively, in astrocytes. Memory consolidation in young chickens requires glycogenolysis (Gibbs et al., 2006; Hertz and Gibbs, 2009). The successful consolidation of memory from short-term to long-term memory requires neuronal NA release (Gibbs et al., 2010). Therefore, it appears that NA, released from neurons, such as those from locus coeruleus, initiates astrocytic morphological changes and activates astroglial energy metabolism. Thus, NA may be considered as an integrator of the metabolism, morphology and function of astrocytes. In the adult operational (i.e., awake) brain, NA is the main signaling molecule that triggers astroglial Ca^2+^ signaling (Ding et al., 2013), which represents the universal form of glial excitability (Verkhratsky et al., 1998).

## Astrocytes as Hubs for the Network Reset System

The LC is the primary source of NA in the CNS. It is localized in the brainstem and projects widely, and is thus able to synchronously activate neural networks in several brain regions. This may be regarded as a functional “reset” for many brain networks (Bouret and Sara, 2005; Sara, 2015). Axons of LC neurons project to the spinal cord, the brain stem, the cerebellum, the hypothalamus, the thalamic relay nuclei, the amygdala, the basal telencephalon, and the cortex, although some cortical areas receive more abundant innervation (Chandler et al., 2014). In all these structures, synchronous activation of LC projections (Bouret and Sara, 2005) leads to coherent and synchronized electrical activity, possibly reflected by gamma waves on an electroencephalogram (Sara, 2015). LC innervation mediates arousal and the sleep–wake cycle, attention and memory, behavioral flexibility, behavioral inhibition and stress, cognitive control, emotions, neuroplasticity, posture, and balance (Benarroch, 2009). The effects of NA are mediated through α- and β-adrenergic receptors (α/βARs) which are expressed in neurons, microglia, and astrocytes. The ARs were among the first receptors to be causally linked to astroglial Ca^2+^ signaling (Salm and McCarthy, 1989; Kirischuk et al., 1996). Increases in astroglial Ca^2+^ were observed *in vivo* after stimulation of the LC in anesthetized animals (Bekar et al., 2008). In awake animals, stimulation of LC neurons triggered (by activation of α-ARs) widespread astroglial Ca^2+^ signals, which appeared in almost all astrocytes in the field of study (Ding et al., 2013). This synchronous response may represent the means by which neural networks are coordinated. Simultaneously, through activation of β-ARs, the cAMP-dependent pathways are activated; this in turn instigates rapid degradation of glycogen, which serves as the main energy reserve in the brain (Prebil et al., 2011; Kreft et al., 2012) and initiates morphological plasticity of astrocytes (Vardjan et al., 2014).

## Vesicular Release of Gliosignaling Molecules

By having secretory vesicles clustered close to the plasma membrane, which is a hallmark of the active zone of the presynaptic terminal, the delay between the incoming stimulus and secretion is minimized, being as short as 100 μs (Sabatini and Regehr, 1999). At the same time, vesicle-based release of chemical messengers can exhibit much longer delays in stimulus–secretion coupling. In astrocytes, the mechanism prolonging the time between the arrival of the stimulus and the release of transmitters has been naturally selected, because the maximal speed of regulated exocytosis in astroglia appears much slower than that in neurons (Guček et al., 2012; Neher, 2012; Zorec et al., 2015). Regulated exocytosis also plays a role in many forms of cell-to-cell communication besides release of transmitters, being for example critical for the delivery of transporters, ion channels and antigen presenting molecules to the cell surface (Guček et al., 2012). Vesicular trafficking and release, which have evolved ~3 billion years ago in arhaea (Spang et al., 2015), is fundamental for signaling and communication within the relatively large eukaryotic cell volume. Communication within large cells could no longer be supported by diffusion-based processes, which provide effective and rapid transport of molecules within the submicron range. Hence the development of subcellular organelles, such as secretory vesicles, presented a solution for the “signaling problem” in the relatively large volume of eukaryotic cells, to which astrocytes belong (Guček et al., 2012).

An ideal approach to monitor the rate-limiting processes of regulated exocytosis in astrocytes at the cellular level is to measure changes in the plasma membrane area, which reflects the fusion of vesicles with the plasma membrane. This can be monitored by measuring membrane capacitance (*C*_m_), which is linearly related to the membrane area (Neher and Marty, 1982). This technique was used in cultured astrocytes (Kreft et al., 2004) to test the hypothesis that an increase in [Ca^2+^]_i_, after photolysis of caged Ca^2+^ (Neher and Zucker, 1993), elicits an increase in the whole-cell *C*_m_. A half-maximal increase in *C*_m_ of these astrocytes was attained at ~27 μM [Ca^2+^]_i_, which is similar to the Ca^2+^-dependency of regulated exocytosis in various types of neurons, recorded by a similar technique (Heidelberger et al., 1994; Bollmann et al., 2000; Kreft et al., 2003a). In contrast to neurons, however, a rather small, within 100 nM, increase in [Ca^2+^]_i_ from the resting level was sufficient to induce glutamate release from astrocytes, as detected by glutamatergic effects on nearby neurons, used as sniffer cells (Parpura and Haydon, 2000). A similar high-affinity Ca^2+^ sensing mechanism for vesicular release was reported in pituitary endocrine cells (Kreft et al., 2003b). At present, astrocytes appear to be the slowest secretors of all the excitable mammalian cells investigated thus far. The kinetics of *C*_m_ increase is at least two orders of magnitude slower than the kinetics of regulated exocytosis recorded by a similar technique in neurons (Kreft et al., 2004; Neher, 2012). The Ca^2+^-dependent increases in *C*_m_ were sensitive to tetanus toxin (which cleaves synaptobrevin 2 and cellubrevin), indicating a soluble *N*-ethyl maleimide-sensitive fusion protein attachment protein receptor and Soluble NSF Attachment Protein Receptor (SNAP)-based vesicular mechanism (Kreft et al., 2004).

Why is regulated exocytosis in astrocytes so slow? One reason is the distinct slow kinetics of molecular mechanisms regulating the vesicle membrane–plasmalemma merger. The number of SNARE molecules per vesicle, which is relatively low in astrocytes (Singh et al., 2014), may also contribute to the slow kinetics of regulated exocytosis. Slow delivery of vesicles to the plasma membrane fusion sites may also play a significant role. The vesicle dynamics is an amazingly elaborate system, regulated by increases in [Ca^2+^]_i_ (Potokar et al., 2013; Vardjan et al., 2015). For example, the complexity of vesicle traffic regulation in astrocytes is characterized by two typical, yet opposing, properties of vesicles that contain peptides, such as atrial natriuretic peptide, and/or ATP, and those that carry amino acids, such as glutamate and D-serine, and are labeled by the glutamate transporter VGLUT1 (Potokar et al., 2005, 2013; Vardjan et al., 2012; Vardjan and Zorec, 2015). Glutamatergic vesicles speed up with an increase in [Ca^2+^]_i_ (Stenovec, 2007), whereas the same increase in [Ca^2+^]_i_ slows down peptidergic vesicles and endolysosomes (Potokar et al., 2010).

Glutamatergic and peptidergic vesicles have the capacity to recycle. The mobility of recycling peptidergic vesicles was studied in cultured astrocytes (Potokar et al., 2008) and in intact brain slices (Potokar et al., 2009). At rest, peptidergic vesicles moved faster and more directionally than after the exposure of astrocytes to ionomycin to increase [Ca^2+^]_i_ (Potokar et al., 2008). The effect of increased [Ca^2+^]_i_ was dramatic; the movement of vesicles was almost halted, with only a jitter associated with random diffusional movement remaining. At least some of the peptidergic vesicles carry ATP and a similar attenuation was observed in their mobility when astrocytes were stimulated (Pangrsic et al., 2007).

What is the physiologic significance of differential mobility of vesicles carrying specific cargo, for example, classic chemical transmitter vs. neuromodulators or neuropeptides? An increase or decrease in vesicle mobility may affect the efficiency of vesicle merger with the plasma membrane and the subsequent cargo discharge. It is possible that vesicles engaged in the dichotomous regulation of vesicle traffic exhibit different vesicle sizes, which may determine the nature of vesicle traffic and fusion with the plasmalemma, as was reported for endocrine cells (Flašker et al., 2013). Increased mobility of glutamatergic vesicles (which can quickly refill using VGLUTs) may indicate that they could be discharged at multiple loci at times of increased Ca^2+^ excitability, resulting in more diffuse signaling as opposed to spatially precise information transfer so characteristic of neuronal synaptic transmission. This speculation seems to be aligned with the ability of astrocytes to modulate synaptic transmission at a long temporal domain and via broad extrasynaptic access sites to neurons.

Impaired astrocytic vesicle traffic has been tentatively associated with intellectual deficiency (ID). Symptoms of ID appear early in life and the disease affects about 2% of the population. Family studies have demonstrated a relatively large number of X chromosome-linked forms of ID (XLID) with an incidence of about 0.9–1.4 in 1000 males (Turner, 1996). One of the first genes found to be mutated in patients with XLID is GDP dissociation inhibitor 1 (GDI 1; D’Adamo et al., 1998), which encodes for guanine nucleotide dissociation inhibitor (αGDI), a protein physiologically involved in regulating GDP-bound RAB proteins. The identification of *GDI1* association with ID suggested that vesicular traffic in neural cells is an important pathway for the development of cognitive functions (D’Adamo et al., 2002; Bianchi et al., 2009). Although the importance of αGDI in neuronal function has been demonstrated, it is unclear whether its role in glia vesicle trafficking contributes to the disease. The αGDI protein regulates the function of RAB GTPases, such as RAB 4 and RAB 5, which have been shown to regulate vesicle dynamics in astrocytes (Potokar et al., 2012), and it is likely that impaired vesicle traffic in astrocytes contributes to ID, which is linked to impaired cognitive processes involving memory formation.

## Conclusion

Astroglial cells control homeostasis in the CNS to support many processes including memory formation. Astrocytes contribute to memory as signaling hubs and as structures that alter their morphology and recruit energy resources for memory consolidation. Excitation–secretion coupling in astrocytes is loose and this may be of particular importance to support the slowness of the overall memory-related structural dynamics in the CNS.

## Author Contributions

RZ, AH, NV, AV wrote the manuscript.

## Conflict of Interest Statement

The authors declare that the research was conducted in the absence of any commercial or financial relationships that could be construed as a potential conflict of interest.
